# Comparison of hemostatic efficacy of topical Ankaferd Blood Stopper on heparinized and nonheparinized rats in bleeding related to liver injury

**DOI:** 10.1590/ACB360106

**Published:** 2021-02-01

**Authors:** Metin Ergin, Nazmi Özer

**Affiliations:** 1 MD. Fatih Unıversıty – Faculty of Medicine – Ankara, Turkey.; 2 Associate Professor. University of Health Sciences – Adana City Training and Research Hospital – Department of General Surgery – Adana, Turkey.

**Keywords:** Liver, Hemorrhage, Heparin, Surgical Hemostasis, Rats, Surgicel

## Abstract

**Purpose:**

In this study, hemostatic efficacy of Ankaferd Blood Stopper (ABS), a new generation hemostatic agent, was compared in the presence of heparin effect.

**Methods:**

Forty-eight Wistar albino rats were divided into two main groups as heparinized and nonheparinized, and these two main groupswere divided into six subgroups as control, Surgicel and ABS (n = 8). Grade 2 liver injury was performed on rats as standard. All groups were compared in terms of weight, laceration surface area, prothrombin time (PT), activated partial thromboplastin time (aPTT), international normalized ratio (INR), bleeding time, bleeding amount, hemoglobin (Hb) levels, macroscopic and microscopic reactions to the agent used.

**Results:**

Whereas there was no statistically significant difference between weight, laceration surface area, PT, INR and preoperative Hb values in the heparinized and nonheparinized groups, postoperative Hb, bleeding time, bleeding amount and aPTT values were statistically different (p < 0.05). In the heparin-hemostat interaction, the ABS group had the lowest bleeding in the heparinized group in terms of the amount of bleeding compared to the control and Surgicel groups (F = 0.764; p = 0.047). In macroscopic and microscopic comparison, there was no difference between the groups in terms of cell necrosis andfresh bleeding (p > 0.05), it was found that the Surgicel group had statistical significantly higher reaction scores (p < 0.05) than the other groups in terms of other parameters.

**Conclusions:**

Ankaferd Blood Stopper can be safely and effectively used in surgical practice and in patients with additional diseases requiring heparinization, since it causes minimal reaction in the liver and decreases the amount of bleeding especially in the heparinized group.

## Introduction

The liver is the largest solid organ in the human body and is the most frequently injured organ in abdominal trauma. Except for gunshot and stab injuries, the majority of liver injuries occur as a result of blunt trauma^1^.

Liver injuries resulting from blunt abdominal trauma are more complex than penetrating injuries and mortality rates are higher. Reported mortality rates according to the degree of injury are 7-13% for stage III, 30% for stage IV, and 66-82% for stage V and VI. In stage V and VI injuries, the vast majority of cases die before they have the opportunity to perform any intervention. Mortality rate in major liver surgery is 3-14%, and the most common reason for this is bleeding^1^,^2^.

The only aim of emergency surgery in liver trauma is to stop the bleeding. It is more difficult to control the situation in people who receive anticoagulant therapy, which increases mortality and morbidity. There are many methods available to stop bleeding and one of this method is topical hemostatic agents^3^.

One of these agents is Ankaferd Blood Stopper (ABS), which is a hemostatic agent obtained from five plant extracts. It is generally used in dental bleeding, skin bleeding, intra-abdominal and cardiovascular bleeding^4^. It was observed that it has bacteriostatic effect on gram-positive and gram-negative microorganisms in studies. Therefore, it has positive effects on infection control and wound healing^5^. In addition, it also has antitumoral and antioxidant properties as a feature of the plant extracts in its content^6^,^7^.

When looking at hemostatic effect of this agent, ABS creates a structure network in plasma and serum in a short time. As a result of general hemostasis this structure network is formed by the ABS interaction between proteins and mainly fibrinogen in the blood. The process of stopping the bleeding is mainly dependent on protein agglutination^8^.

This work aimed to compare ABS efficacy with oxidized regenerated cellulose (Surgicel), which is a hemostatic agent, on heparinized and nonheparinized groups, since there is no previous study in the literature on intraabdominal solid organ bleeding who used anticoagulants with ABS before.

## Methods

The study was supported by Fatih University with the project number P 53010804-2 and the approval of the ethics committee dated 06.03.2008 and numbered 23-3868 was obtained in the experimental animal laboratories of Gazi University experimental medicine research center. This study was carried out in accordance with the rules for the care and use of laboratory animals specified in the ARRIVE guideline and the Helsinki declaration.

In this study, 48 male Wistar albino rats weighting 370 g (range 320-420g) were used. The animals were kept in polycarbonate cages and fed rat food (Purina, Nestle Co. USA.) in the form of standard dry pellets. They were kept under 20 ± 2°C room temperature, 50% ± 10 humidity, 12-hour light/dark period. The rats were randomly divided into two equal main groups as heparinized and nonheparinized. Two hours before the operation, 400 U/kg heparin sodium (Nevparin injectable/5mL-Mustafa Nevzat) was administered subcutaneously to one of the main groups (heparinized). Heparin was not given to the second main group (nonheparinized). Hemoglobin (Hb), prothrombin time (PT), activated partial thromboplastin time (aPTT) and international normalized ratio (INR) were measured from the blood taken from the tails of all rats 2 h after heparin administration. These two main groups were divided into three subgroups as control, Surgicel and ABS (n = 8) (Table 1).

**Figure 1:**
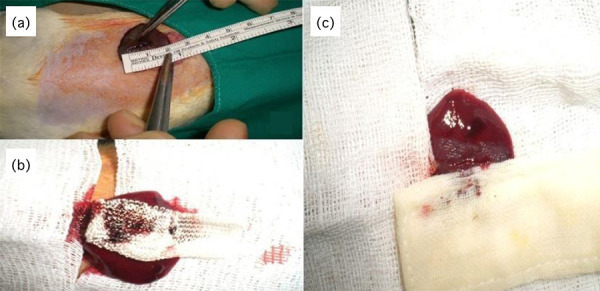
(**a**) Creation of grade 2 liver damage to rats after laparotomy and measurement of laceration area; (**b**) Providing hemostasis with Surgicel; (**c**) Providing hemostasis with ABS.

From these subgroups, grade 2 liver laceration was performed to the control subgroup and compression was applied only with gauze buffer for hemostasis. Surgicel and compression were applied to the Surgicel subgroup after standard injury (Johnson & Johnson Wound Management, Ethicon, Inc. Somerville, NJ-USA). The ABS subgroup was also applied ABS pad and compression after the standard injury (Ankaferd Health Products Co. İstanbul, Türkiye) (Fig. 1).

**Table 1:** Separation of heparinized and nonheparinized groups according to hemostatic agents.

Description		Group name
Group 1		Nonheparinized control
Group 2		Nonheparinized surgicel
Group 3		Nonheparinized ABS
Group 4		Heparinized control
Group 5		Heparinized surgicel
Group 6		Heparinized ABS

### Surgical technique

All animals were anesthetized by intraperitoneal administration of 60 mg/kg ketamine hydrochloride (Ketalar Eczacıbaşı Warner-Lambert pharmaceutical industry, Levent, İstanbul) and 10 mg/kg xylazine hydrochloride (Rompun Bayer, Şişli, İstanbul) under aseptic conditions. The abdominal area of all subject animals, were wiped with 7.5% povidone iodine after shaving and cleansing. After median laparotomy, grade 2 laceration of 10-15 mm in length and 1-1.5 mm in depth was created with a 15-gauge scalpel near the free edge of the left liver lobe in rats. The surface area of laceration performed for all rats was measured and recorded. Compression was used as a standard to achieve hemostasis in all groups. During this application, bleeding was observed at the end of the 30 s compression, if it continued, compression and observation was continued for 30 s until it stopped. The bleeding time during the procedure was recorded by calculating the amount of bleeding in the postoperative period as well. The amount of bleeding was calculated by weighing gauze pads with preoperative and postoperative precision scales (Mettler Toledo AB 204-S-USA).

**Figure 2:**
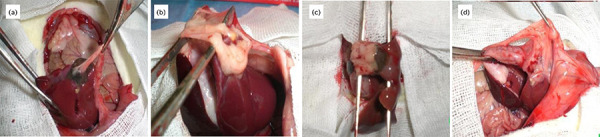
Macroscopic view of the adhesion stages: (**a**) Stage 1 adhesion; (**b**) Stage 2 adhesion; (**c**) Stage 3 adhesion; (**d**) Stage 4 adhesion.

Bleeding time was determined after no bleeding was observed on the surgical surface for 10 min. In all groups, Hb values were determined and recorded in the preoperative and 24 h postoperatively. The adhesions between the liver and other tissues were dissected with relaparotomy following anesthesia on the postoperative 7th day. For these adhesions, evaluation was made with the scale defined by Nair *et al*.^9^ (Table 2) (Fig. 2). At the end of the study, 4-5 mL of intracardiac blood was drawn and rats were sacrificed.

**Table 2:** Adhesion stage scores.

Stage		Description
0		No adhesion
1		Only one adhesion band between the organs
2		Two adhesion bands between the organs or between one organ and abdominal wall
3		More than 2 adhesion bands between the organs
4		Adhesion of all viscera to the abdominal wall

### Biochemical analysis

Prothrombin time, aPTT and INR, preoperative and postoperative Hb values were obtained from blood samples. Prothrombin time test measures how long it takes for a clot to form in a blood sample; and it was determined by coagulometric method using Thromborel S (Dade Behring OUHP G29) kit, at 405 nm wavelength with an optical reader. Activated partial thromboplastin time was determined using Pathromtin SL (Dade Behring OQGS G17) kit, coagulometric method, at 405 nm wavelength with an optical reader^10^,^11^. An INR is a type of calculation based on PT test results. Hemoglobin was detected in EDTA tubes by using blood counter (Beckman Coulter HMX California/USA) device.

### Histopathological analysis

**Figure 3:**
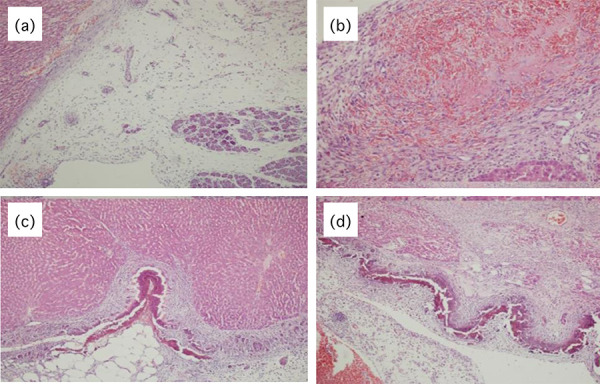
Histopathological examination of tissues (H&Estaining 200×). (**a**) Omental tissue and pancreas (adjacent organ) adhesion formation; (**b**) Fresh bleeding areas; (**c**) Mixed inflammatory cell infiltration; (**d**) Moderate fibrosis areas.

After taking 5-7 μm sections from the liver, which was fixed in 10% formaldehyde for 24 h and impregnated with paraffin, these sections were stained with hematoxylin-eosin. They were examined at 200 magnification under a light microscope. The samples were evaluated histopathologically by a single pathologist who was unaware of the tissues. The histopathological evaluation (Fig. 3) used the parameters presented in Table 3.

**Table 3:** Histopathological evaluation criteria.

**Score**	**Inflammation localization**	**Inflammation density**	**Fibrosis**	**Omental adhesion**	**Foreign body reaction**	**Fresh bleeding**	**Cell necrosis**
0	No inflammation	No inflammation	No	No	No	No	No
1	Limited in capsule	Mild lymphocyte-plasma cell infiltration	Minimal loose fibrosis	Adhesion between liver lobes	Mild	Focal focus, small	Focal, in capsule
2	Capsules + a few periportal - mildly severe lymphocytes in the portal area	Moderate lymphocyte-plasma, neutrophil leukocyte, histiocyte infiltration	Moderate fibrosis	Omental adhesion, mild inflammation	Moderate	Multi focal focus	Multifocal in the parenchyma, small foci
3	Moderate - severe in the periportal - portal area	Severe mixed inflammation or microabscess	Dense fibrosis	Omental adhesion, severe inflammation, fibrosis	Severe	Massive	In parenchyma, large
4	-	Widespread abscess formation	-	Adhesion to neighboring organs and structures	-	-	-

### Statistical analysis

SPSS 21 program were used for statistical analysis. The compliance of the data to normal distribution was checked graphically and with the Shapiro-Wilk test. Various transformation methods were applied for parameters that did not conform to the normal distribution, and their suitability to normal distribution was checked again. Student’s t-test and Mann-Whitney U test were used in the comparison of the two groups. For comparison of more than two groups, analysis of variance (ANOVA) or Kruskal-Wallis variance analysis, which is its nonparametric counterpart, was used. Bonferroni post-hoc test orMann-Whitney U test with Bonferroni correction was used in order to determine the source of the difference in case of a difference as a result of variance analysis. Multidirectional analysis of variance was used to examine the effects of variables on clinical parameters. Fisher LSD post-hoc test was applied to find out. P < 0.05 was considered significant in statistical comparisons.

## Results

Rats were randomly distributed evenly into nonheparinized and heparinized groups. Among the clinical parameters, postoperative Hb value showed a statistically significant difference between heparinized and nonheparinized groups (t = 2.303; p = 0.032). Postoperative Hb value is higher in nonheparinized subjects than heparinized subjects. Bleeding time value is lower in nonheparinized subjects compared to heparinized subjects and the difference is statistically significant(t = 7.351; p < 0.001).

The value of the aPTT parameter was statistically significantly higher in the heparinized group compared to the nonheparinized group (p < 0.001). There was no statistically significant difference between heparinized and nonheparinized groups in terms of weight, laceration surface area, preoperative Hb value, PT and INR values (p > 0.05). The amount of bleeding was statistically significantly higher in the heparinized group compared to the nonheparinized group (t = 2.303;p < 0.026) (Table 4).

**Table 4:** Comparison of clinical parameters according to heparinized and nonheparinized groups.

**Parameter**	**Group**	**n**	**Average**	**Std. deviation**	**t**	**p**
Weight	Nonheparinized	24	354.48	30.37	0.032	0.974
	Heparinized	24	354.22	26.73		
Surface area	Nonheparinized	24	2.42	0.15	0.106	0.916
	Heparinized	24	2.42	0.15		
Hb preop	Nonheparinized	24	15.70	1.48	1.949	0.058
	Heparinized	24	14.90	1.32		
Hb postop	Nonheparinized	24	12.83	1.63	2.206	0.032
	Heparinized	24	11.83	1.52		
Bleeding time	Nonheparinized	24	1.33	0.25	7.351	< 0.001
	Heparinized	24	1.94	0.31		
Bleeding amount	Nonheparinized	24	1.55	0.97	2.303	0.026
	Heparinized	24	2.14	0.80		
PT	Nonheparinized	24	13.70	1.65	1.061	0.294
	Heparinized	24	14.22	1.80		
INR	Nonheparinized	24	1.00	0.12	1.063	0.293
	Heparinized	24	1.04	0.14		
Parameter	Group	n	Median	Rank	Z	p
aPTT	Nonheparinized	24	20.00	12.50	5.399	< 0.001
	Heparinized	24	62.95	36.50		

Since the aPTT parameter does not conform to the normal distribution, the Mann–Whitney U test, which is the nonparametric equivalent of the Student’s t-test, was used in paired comparisons. For non-aPTT parameters: Student’s t-test; for aPTT: Mann–Whitney U test.

As a result of the comparison of clinical parameters (weight, surface area, preoperative and postoperative Hb level, bleeding time, bleeding amount, PT, INR) according to hemostats, no statistically significant difference was found (p > 0.05) (Table 5).

**Table 5:** Comparison of clinical parameters with analysis of variance according to hemostats (ANOVA).

**Parameter**	**Hemostat**	**n**	**Average**	**Std. deviation**	**F**	**p**
Weight	Control	16	354.12	33.29	0.366	0.696
	Surgicel	16	350.13	24.71		
	ABS	16	358.80	27.41		
Surface area	Control	16	2.39	0.14	1.044	0.360
	Surgicel	16	2.40	0.14		
	ABS	16	2.46	0.17		
Hb preop	Control	16	15.23	1.32	0.081	0.922
	Surgicel	16	15.43	1.83		
	ABS	16	15.28	1.15		
Hb postop	Control	16	12.31	1.57	0.004	0.996
	Surgicel	16	12.36	2.03		
	ABS	16	12.33	1.35		
Bleeding time	Control	16	1.65	0.45	0.067	0.935
	Surgicel	16	1.60	0.41		
	ABS	16	1.65	0.42		
Bleeding amount	Control	16	1.44	0.73	2.541	0.090
	Surgicel	16	1.97	1.03		
	ABS	16	2.12	0.91		
PT	Control	16	14.23	1.76	0.349	0.349
	Surgicel	16	13.71	1.75		
	ABS	16	13.94	1.75		
INR	Control	16	1.04	0.13	0.349	0.707
	Surgicel	16	1.00	0.13		
	ABS	16	1.02	0.13		

When the subjects were divided into two separate groups as heparinized and nonheparinized, the change of clinical parameters between subgroups (control, Surgicel, ABS) was examined.

There was no statistically significant difference in heparin-hemostat interaction on weight, preoperative Hb and postoperative Hb levels, laceration surface area, bleeding time, aPTT parameters (p > 0.05).

Heparin-hemostat interaction creates a statistically significant difference on the amount of bleeding (F = 0.764; p = 0.047). According to Fisher LSD post-hoc test results; there is a statistically significant difference between the mean amount of bleeding between the subjects in group 6 and the subjects in group 4 (p < 0.001) and group 5 (p = 0.008) (Table 6).

**Table 6:** Variance analysis of clinical parameters according to heparinized and nonheparinized hemostats.

**Group**	**Parameter**	**Hemostat**	**n**	**Average**	**Std. deviation**	**F**	**p**
Nonheparinized	Weight	Control	8	341.33	26.03	1.871	0.179
		Surgicel	8	352.65	33.14		
		ABS	8	369.48	28.24		
	Surface area	Control	8	2.38	0.14	0.560	0.579
		Surgicel	8	2.42	0.15		
		ABS	8	2.46	0.17		
	Hb preop	Control	8	16.05	1.33	0.262	1.772
		Surgicel	8	15.91	1.84		
		ABS	8	15.56	0.80		
	Hb postop	Control	8	12.90	0.98	0.124	0.884
		Surgicel	8	12.58	1.47		
		ABS	8	12.78	1.44		
	Bleeding time	Control	8	1.27	0.28	0.619	0.548
		Surgicel	8	1.41	0.23		
		ABS	8	1.31	0.26		
	Bleeding amount	Control	8	2.31	0.82	0.404	0.673
		Surgicel	8	2.01	0.62		
		ABS	8	2.01	0.84		
	PT	Control	8	13.92	2.00	0.121	0.887
		Surgicel	8	13.50	1.51		
		ABS	8	13.66	1.61		
	INR	Control	8	1.02	0.15	0.122	0.886
		Surgicel	8	0.99	0.11		
		ABS	8	1.00	0.12		
**Group**	**Parameter**	**Hemostat**	**n**	**Average**	**Std. deviation**	**F**	**p**
Heparinized	Weight	Control	8	366.91	36.38	1.402	0.268
		Surgicel	8	347.61	13.99		
		ABS	8	348.13	23.50		
	Surface area	Control	8	2.41	0.14	0.608	0.554
		Surgicel	8	2.38	0.14		
		ABS	8	2.46	0.17		
	Hb preop	Control	8	16.03	0.67	0.361	0.701
		Surgicel	8	15.94	1.35		
		ABS	8	15.61	0.93		
	Hb postop	Control	8	12.46	0.94	0.003	0.997
		Surgicel	8	12.51	1.93		
		ABS	8	12.51	1.07		
	Bleeding time	Control	8	2.03	0.13	0.271	0.765
		Surgicel	8	2.06	0.27		
		ABS	8	1.98	0.22		
	Bleeding amount	Control	8	2.72	0.56	0.764	0.047
		Surgicel	8	2.35	0.82		
		ABS	8	2.24	1.02		
	PT	Control	8	14.53	1.57	0.217	0.806
		Surgicel	8	13.92	2.04		
		ABS	8	14.22	1.96		
	INR	Control	8	1.06	0.12	0.217	0.807
		Surgicel	8	1.02	0.15		
		ABS	8	1.04	0.15		
**Group (aPTT)**		**Hemostat**	**n**	**Median**	**Average Rank**	**X2**	**p**
Nonheparinized Surgicel ABS		Control	8	61.30	12.50	0.061	0.970
		8	60.85	12.94			
		8	63.30	12.06			
Heparinized Surgicel ABS		Control	8	19.95	12.38	0.065	0.968
		8	19.70	12.13			
		8	20.00	13.00			

Kruskal–Wallis analysis of variance. ANOVA analysis of variance was used for non-aPTT parameters, since the aPTT did not conform to the normal distribution.

Macroscopic adhesion scores were not statistically different between heparinized and nonheparinized groups (t = 0.614; p = 0.438). Macroscopic adhesion scores were statistically different between hemostats (F = 6.853; p = 0.003). There was a statistically significant difference between the control and Surgicel groups in terms of macroscopic adhesion scores (p = 0.013). The Surgicel group has a higher macroscopic adhesion score than the control group. Similarly, there is a statistically significant difference between ABS and Surgicel groups in terms of macroscopic adhesion score (p = 0.001). The Surgicel hemostat macroscopic adhesion score is higher than that of the ABS hemostat. While Surgicel hemostat has the highest macroscopic adhesion score among groups, ABS hemostat has the lowest macroscopic adhesion score.

## In the examination of tissue sampleswith a light microscope

When the localization of inflammation was examined, it was examined according to heparinization, hemostats and groups. Inflammation localization scores were similar in heparinized or nonheparinized rats (Z = 0.066; p = 0.947). Similar to heparinization, inflammation localization scores between control, Surgicel and ABS hemostats were also not statistically significant (X^2^ = 1.887; p = 0.389).

When examined in terms of inflammation intensity scores; there was no statistically significant difference between heparinized and nonheparinized groups (Z = 0.034; p = 0.973). In control, Surgicel and ABS groups, the inflammation intensity scores are statistically significantly different (X^2^ = 12.342; p = 0.002). In Surgicel, the inflammation intensity score is higher than in control. Similarly, Surgicel inflammation intensity score (as in the control) is higher than ABS (Z = 3.387; p < 0.001). Surgicel has the highest inflammation intensity score among hemostats, while ABS hemostat has the lowest inflammation intensity score.

When examined in terms of fibrosis; fibrosis scores did not show statistically significant difference between heparinized and nonheparinized subjects (Z = 0.917; p = 0.359). The fibrosis scores were statistically significantly different between hemostats (X^2^ = 14.551; p = 0.001). Fibrosis score was higher in Surgicel group when compared to control group (p < 0.001). Similarly, Surgicel group score was higher than ABS’s (p = 0.009). Among the hemostats, while the highest fibrosis score among hemostats was seen in the Surgicel group, the control group had the lowest fibrosis score compared to all groups.

When examined in terms of omental adhesion scores, there was no statistically significant difference between heparinized and nonheparinized groups (Z = 1.003;p = 0.316). Adhesion scores between hemostats were statistically significantly different (X^2^ = 15.017; p = 0.001). Adhesion score was higher in Surgicel group compared to control group (Z = 3.037; p = 0.002). Adhesion score was higher in Surgicel group than ABS group (Z = 2621; p = 0.009). While the highest omental adhesion score among hemostats was observed in Surgicel group, control and ABS groups had almost equal adhesion scores.

When examined in terms of foreign body reaction; there was no statistically significant difference between heparinized and nonheparinized groups (Z = 0.166; p = 0.868). Foreign body reaction scores were statistically significantly different between hemostats (X^2^ = 28.986; p < 0.001). Foreign body reaction score was higher in Surgicel group compared to control group (Z = 4.5542; p < 0.001). Similarly, Surgicel foreign body reaction score was higher than that of ABS group (Z = 4.795; p < 0.001). While the highest foreign body reaction score among hemostats is observed in Surgicel group, the control group has the lowest foreign body reaction score.

When examined in terms of cell necrosis and fresh bleeding scores, there was no statistically significant difference between heparinized and nonheparinized groups and between hemostats in terms of cell necrosis and fresh bleeding scores (p > 0.05).

## Discussion

Many methods have been tried to prevent liver parenchymal bleeding depending on the cause of the bleeding. Among these methods, there are also the use of hemostatic materials^12^-^14^. There are studies on hemostats in normal bleeding, but there was no study with hemostats on intraabdominal solid organ bleeding in heparinized patients.

The basic mechanism of action of most of the local hemostatic agents is to ensure the secretion of mediators that provide natural hemostasis by activating thrombocytes. Some substances also have auxiliary mechanisms of action. For example, fibrin preparations have an adhesive effect and bovine collagen has gag-forming effects^15^.

Surgicel (oxidized regenerated cellulose) is used in the form of gauze or cotton pads. Where they are applied, they swell by drawing water and transform into cellulosic acid to form an artificial clot. In addition to its local hemostatic properties, it has been shown to be bactericidal *in vitro* against aerobic, anaerobic, gram-positive and gram-negative organism species^16^. They are compounds that swell after contact with liquids and act to effectively fill the wound area. They show better hemostatic tendency on surfaces that are relatively blood free. However, adhesion and coagulation character are generally not good. Recently, concerns have arisen about the complications arising from the material getting out of the wound area during spine surgery^17^. Also, due to the granulomatous reaction it caused, it resulted in an appearance that could be confused with recurrence in computed tomography controls^18^.

Ankaferd Blood Stopper is a mixture of five herbal extracts standardized in certain proportions. Each of them has hematological and vascular effects. *Glycyrrhiza glabra* has anti-inflammatory, antithrombin, antithrombocyte, antioxidant, antiatherosclerotic and antitumor effects^6^. It inhibits angiogenesis, decreases vascular endothelial growth level and cytokine-dependent neovascularization^19^. Besides its antibacterial effect, *Thymus vulgaris* has a protective effect from lipid peroxidation due to its antioxidant effect^20^. *Vitis vinifera* has antioxidant and antitumoral effect^7^. *Alpinia officinarum* provides cyclooxygenase enzyme increase^21^. *Urtica dioica* increases nitric oxide production in vascular endothelium, causing vasodilation^22^.

Ankaferd Blood Stopper creates a structure network in plasma and serum in a short time. As a result of general hemostasis and biochemical tests, it has been revealed that this structure network was formed by the mutual effect that ABS has with proteins and mainly with fibrinogen in the blood. It has been observed that ABS does not affect factors II, V, VII, VIII, IX, X, XI and XIII in plasma^23^. Plasma fibrinogen activity and consequently thrombin time were prolonged. In addition, total protein, albumin and globulin levels decreased significantly following ABS application. Blood arrest is basically dependent on protein agglutination. In the ABS network, the physiological hemostatic process develops independently of the individual blood coagulation structure (without affecting it). Therefore, ABS is effective both in normal individuals and individuals with impaired primary or secondary hemostasis^24^.

Heparin is a sulfated polysaccharide (glycosaminoglycan) mixture, it has an anticoagulant effect by activating the antithrombin III (AIII) in the blood, which is synthesized in the liver dependent on vitamin K. Activated AIII disrupts coagulation by inhibiting thrombin, coagulation factors (XIIa, XIa, Xa, Xa) and kallikrein in blood circulation. It can increase or decrease the aggregation depending on the conditions.

In this study, it was determined that there was no statistically significant difference between groups and subgroups in weight, laceration surface area, preoperative Hb, PT and INR values, and bleeding time and aPTT were long in the heparinized group. In terms of the amount of bleeding, a significantly lower amount of bleeding was observed in the ABS group in the heparinized groups compared to the control and Surgicel groups.

While the heparinization procedure did not create a statistically significant difference in terms of macroscopic adhesion, when looked at hemostats; the degree of macroscopic adhesion was higher in the Surgicel group compared to the control and ABS groups.

In terms of heparinization, when the location of inflammation was evaluated, there was no difference. In the hemostats, inflammation intensity was higher in the Surgicel group compared to the control and ABS groups. Although the heparinization procedure does not cause a statistically significant difference in terms of fibrosis, in hemostats, fibrosis was higher in the Surgicel group compared to control and ABS groups.

While there was no statistically significant difference in omental adhesion between the nonheparinized and heparinized groups, omental adhesion was higher in the Surgicel group compared to the control and ABS groups. While heparinization did not make a statistically significant difference in terms of foreign body reaction, when looking at the hemostats, the foreign body reaction in the Surgicel group was significantly higher than in the ABS and control groups. While heparinization did not cause a statistically significant difference in terms of cell necrosis and fresh bleeding, when looking at hemostats, there was no statistically significant difference between control, ABS and Surgicel subgroups in terms of cell necrosis and fresh bleeding.

Considering the literature review, in the study of Akpınar *et al*.^4^, patients who underwent coronary bypass surgery while using clopidogrel and acetyl salicylic acid were divided into two groups. During the operation, ABS was used as a hemostat in the first group, and no hemostat was used in the second group. It was observed that the amount of bleeding and the need for transfusion were significantly lower in the group using ABS compared to the second group. In the study of Akbulut *et al*.^5^, 48 rats were divided into 6 subgroups, heparin was administered to 2 groups and warfarin was administered to 2 groups and nothing to 2 groups, later these rats were tooth extracted and ABS was applied to 3 subgroups for hemostasis (control-ABS, heparin-ABS, warfarin-ABS). Bleeding time was longer in the groups that did not apply ABS, while the amount of bleeding was less in the ABS applied groups. The study by Aydin *et al.* 25 compared calcium alginate and ABS hemostatic activity on 39 rats, and they observed that both calcium alginate and ABS have hemostatic effects in preventing hepatic parenchymal bleeding, ABS causes focal necrosis areas and calcium alginate causes fibrosis. When the hemostatic efficiency of adrenaline and ABS was compared on 20 patients requiring orthodontic intervention, bleeding time was found to be significantly shorter in the ABS group compared to the adrenaline group^26^. Aktop *et al*.^27^, in a study in which they evaluated tissue healing, ABS was showed a positive effect on short-term tissue healing by increasing superoxide dismutase (SOD) activity in rats treated with warfarin. In comparison of ABS and Surgicel in subjects with experimental liver laceration; rats in the ABS and Surgicel groups survived significantly longer than rats in the control group. There was no significant difference between ABS and Surgicel groups in terms of survival^28^. Again, in another study comparing Surgicel and ABS, the latter was found to be more effective in achieving hemostasis and reducing blood loss compared to Surgicel and control groups, and had more encouraging histopathological changes and better intraabdominal adhesion scores^29^.

As can be seen in the literature review, there are no studies on heparinized patients with ABS in intraabdominal solid organ bleeding, and the results of this study will guide the treatment strategy of this patient group in cases of bleeding.

## Conclusions

Ankaferd Blood Stopper causes less fibrosis, inflammation density, adhesion and foreign body reaction compared to Surgicel, and it has been observed that it has a better score than Surgicel in macroscopic adhesion scoring. Due to the fact that the amount of bleeding is significantly less in heparinized patients compared to other groups, Ankaferd Blood Stopper can be used more safely and effectively in surgical practice and in patients with additional diseases requiring heparinization compared to current hemostatic agents.
